# NAD(P)H:Quinone Oxidoreductase-1 Expression Sensitizes Malignant Melanoma Cells to the HSP90 Inhibitor 17-AAG

**DOI:** 10.1371/journal.pone.0153181

**Published:** 2016-04-05

**Authors:** Shuya Kasai, Nobuyuki Arakawa, Ayaka Okubo, Wataru Shigeeda, Shinji Yasuhira, Tomoyuki Masuda, Toshihide Akasaka, Masahiko Shibazaki, Chihaya Maesawa

**Affiliations:** 1 Department of Tumor Biology, Institute of Biomedical Science, Iwate Medical University, Iwate, Japan; 2 Department of Dermatology, Iwate Medical University, Iwate, Japan; 3 Department of Pathology, School of Medicine, Iwate Medical University, Iwate, Japan; University of Alabama at Birmingham, UNITED STATES

## Abstract

The KEAP1-NRF2 pathway regulates cellular redox homeostasis by transcriptional induction of genes associated with antioxidant synthesis and detoxification in response to oxidative stress. Previously, we reported that KEAP1 mutation elicits constitutive NRF2 activation and resistance to cisplatin (CDDP) and dacarbazine (DTIC) in human melanomas. The present study was conducted to clarify whether an HSP90 inhibitor, 17-AAG, efficiently eliminates melanoma with KEAP1 mutation, as the NRF2 target gene, NQO1, is a key enzyme in 17-AAG bioactivation. In melanoma and non-small cell lung carcinoma cell lines with or without KEAP1 mutations, NQO1 expression and 17-AAG sensitivity are inversely correlated. NQO1 is highly expressed in normal melanocytes and in several melanoma cell lines despite the presence of wild-type KEAP1, and the NQO1 expression is dependent on NRF2 activation. Because either CDDP or DTIC produces reactive oxygen species that activate NRF2, we determined whether these agents would sensitize NQO1-low melanoma cells to 17-AAG. Synergistic cytotoxicity of the 17-AAG and CDDP combination was detected in four out of five NQO1-low cell lines, but not in the cell line with KEAP1 mutation. These data indicate that 17-AAG could be a potential chemotherapeutic agent for melanoma with KEAP1 mutation or NQO1 expression.

## Introduction

NRF2 is an oxidative stress-activated transcription factor that regulates transcription of a subset of genes including those encoding enzymes involved in antioxidant synthesis and detoxification [1,2]. Under normal conditions, KEAP1 interacts with NRF2 and E3 ubiquitin ligase CUL3, facilitating NRF2 degradation through the ubiquitin-proteasomal pathway. Oxidation or electrophilic adduction of KEAP1 results in accumulation of NRF2 and its translocation into the nucleus. NRF2 induces transcriptional activation of a number of genes such as those for glutamate-cysteine ligase, which has a role in glutathione synthesis, and NAD(P)H:quinone oxidoreductase-1 (NQO1), which mediates detoxification of endogenous and exogenous oxidants. Although the KEAP1-NRF2 pathway suppresses tumor initiation by attenuating DNA oxidation and electrophilic modification [3], NRF2 has an opposite role in tumor promotion. A number of somatic mutations, or alterations of epigenetic regulation that activates the KEAP1-NRF2 pathway, have been reported in several human malignancies such as non-small cell lung carcinoma (NSCLC), and cancers of the skin and esophagus [4,5]. NRF2 promotes tumor growth through transcriptional activation of genes that shift the glucose and glutamine metabolic pathways to an anabolic direction [6]. In addition, NRF2 activation and elevated levels of antioxidant confer resistance to reactive oxygen species (ROS) produced by chemotherapeutic agents or ionizing radiation [7–9]. Previously, we reported the presence of frame shift mutations in the KEAP1 gene and accumulation of NRF2 in melanoma tissues and melanoma cell lines [10]. Activation of NRF2 increases the concentration of antioxidant and confers resistance to either dacarbazine (DTIC) or cisplatin (CDDP). NRF2 activation also induces the expression of NQO1, which is a key enzyme for bioactivation of quinone-containing chemotherapeutic agents, such as geldanamycin, mitomycin C and β-lapachone [11]. Therefore, these antitumor drugs are potential candidates for the treatment of melanoma that is resistant to dacarbazine or radiotherapy. 17-Allylamino-17-demethoxygeldanamycin (17-AAG, tanespimycin) is a clinically applicable derivative of geldanamycin. 17-AAG inhibits HSP90 chaperone activity and destabilizes its client proteins including melanoma-associated oncogene products, mutated BRAF and AKT [12–14]. Phase I/II clinical trials have reported that 17-AAG was partially effective against malignant melanoma after single administration or in combination with sorafenib or docetaxel, and that the effectiveness was independent of the oncogenic mutation status of patients [15–20].

The present study was conducted to investigate whether melanoma and NSCLC cell lines harboring KEAP1 mutation would be sensitive to 17-AAG. NQO1 was found to be highly expressed in normal melanocytes and several melanoma cell lines, irrespective of the presence of wild-type KEAP1, and they were also 17-AAG-sensitive in comparison with NQO1-low cell lines.

## Materials and methods

### 1. Cell culture

Four human melanoma cell lines (C32, G-361, HMV-II, and SK-MEL-28) were obtained from the Cell Resource Center for Biomedical Research, Tohoku University (Sendai, Japan). Two human melanoma cell lines (A7 and MM-AN) were kindly provided by Dr. M.C. Mihm (Department of Dermatology, Harvard Medical School, Boston, MA). The cells were maintained at 37°C under 5% CO_2_ in RPMI 1640 (Invitrogen, Carlsbad, CA) supplemented with 10% fetal bovine serum (FBS), non-essential amino acids (NEAA) and penicillin-streptomycin (Invitrogen). Two human melanoma cell lines (GAK and HMY-I) were obtained from the Japanese Collection of Research Bioresources (Osaka, Japan) and maintained in F-12 HAM (Sigma Aldrich, St Louis, MO) and DMEM (Invitrogen), respectively, supplemented with 10% FBS, NEAA and antibiotics. Three human melanoma cell lines (MeWo, SK-MEL-2, SK-MEL-31) were obtained from American Tissue Culture Collection (ATCC, Manassas, VA) and maintained according to the culture method of the ATCC. Nine non-small cell lung cancer cell lines (A549, H441, H460, H1299, H1650, H1975, Calu-1, Calu-6, and SK-MES-1) were obtained from the ATCC. All NSCLC cell lines were maintained in RPMI supplemented with 10% FBS, NEAA and antibiotics. Normal human epithelial melanocytes, neonatal (HEMn-LP, HEMn-MP, HEMn-DP) and normal human dermal fibroblasts, and neonatal skin fibroblasts (NHDF-neo) were obtained from Invitrogen and from Lonza (Walkersville, MD), respectively, and maintained in accordance with the supplier’s instructions.

To establish immortalized melanocytes, a human TERT expression vector was constructed by transferring the insert of pBABE-neo-hTERT (Addgene, Cambridge, MA) into the multicloning site of pLVSIN-neo (Takara Bio Inc, Shiga, Japan). Transfection and packaging were carried out using the Lenti-X HTX Packaging system (Takara Bio Inc,) in accordance with the manufacturer’s instructions. HEMn-LP cells were infected and selected in the presence of 800 μg/ml G418 for a week.

### 2. siRNA transfection

Silencer® Select siRNAs against NRF2 (Cat# 4392520, ID s9491 and s9492) and negative control siRNA (Cat# 4390844), Lipofectamine RNAiMAX Transfection Reagent, and Opti-MEM were obtained from Life Technologies (Gaithersburg, MD). Cells were treated with 10 nM siRNA and 7.5 μl RNAiMAX in Opti-MEM in a 6-well plate format in accordance with the manufacturer’s instructions.

### 3. Reagents and antibodies

17-(Allylamino)-17-demethoxygeldanamycin (17-AAG), the NQO1 inhibitor ES936, cis-diammineplatinum (II) dichloride (CDDP), and dacarbazine (DTIC) were obtained from Sigma Aldrich. 17-AAG and ES936 were prepared at a stock concentration of 10 mM in dimethyl sulfoxide. CDDP was directly dissolved in culture medium. DTIC was prepared as a 0.1 M stock in 0.1 M HCl.

The antibody against NRF2 was obtained from Abcam (ab-62352, Cambridge, MA). The antibody against NQO1 was from Cell Signaling Technology (#3187, Boston, MA). α-tubulin antibody was from Sigma Aldrich (T5168). GAPDH antibody was from Covance (MMS-580S, Princeton, NJ). HRP-linked anti-mouse IgG and anti-rabbit IgG were from GE Healthcare (Waukesha, WI).

### 4. Cell viability assay

Trypsinized cells were inoculated into 96-well plates at a density of 3,000 cell/well for 24 h. For dose-response analysis, cells were treated with two-fold serial dilutions of 17-AAG (10 μM to 1 nM), CDDP (1 mM to 0.1 μM) or DTIC (5 mM to 1 μM) for 72 h. NQO1 was inhibited by pretreatment with 100 nM ES936 or vehicle for 30 min, and then an equal volume of 2× concentrated 17-AAG with 100 nM ES936 was added [21]. For combination index calculation, cells were treated with serial dilutions of 17-AAG and CDDP in a molar ratio of 1:100, or 17-AAG and DTIC in a molar ratio of 1:1,000, and analyzed according to the Chou-Talalay method [22]. Cell viability was determined using a Cell Counting Kit-8 (Dojindo, Kumamoto, Japan) in accordance with the supplier’s instructions. Absorbance at 450 nm was measured using a Multiskan Spectrum (Thermo Fisher, Waltham, MA).

### 5. Immunoblotting

Cells were washed twice with cold PBS, harvested by scraping, and lysed in RIPA buffer (50 mM Tris pH 8.0, 150 mM NaCl, 10 mM NaF, 2 mM Na_3_VO_4_, 1% NP-40, 0.5% sodium deoxycholate, 0.1% SDS) supplemented with complete protease inhibitor cocktail (EDTA-free; Roche, Mannheim, Germany) and 0.5 mM PMSF. Protein concentration was measured with a BCA Protein Assay Kit (Novagen, Madison, WI). Total protein lysates of human adult normal tissues (skin, liver and heart) were obtained from BioChain Institute (Newark, CA). Protein samples were separated on SDS-PAGE gel and then transferred onto polyvinylidene difluoride transfer membranes (Pall Corporation, Portsmouth, UK). The membranes were blocked with 5% BSA (Sigma Aldrich) in 0.1% Tween-20/PBS for NQO1 or with 5% non-fat dried milk (#9999, Cell Signaling Technology, Beverly, MA) in 0.1% Tween-20/PBS for other antibodies. The membranes were then immunoreacted with an appropriate primary antibody overnight at 4°C and with HRP-conjugated secondary antibodies (GE Healthcare) for 1 h at room temperature. Signals were detected with ECL prime detection reagents (GE Healthcare) and ChemiDoc XRS (Bio-Rad Laboratories, Hercules, CA). Densitometric analysis of each protein signal was carried out by ImageJ.

### 6. RT-PCR

Total RNA was extracted by using TRIzol Reagent (Life Technologies) in accordance with the manufacturer’s instructions. RNA concentration was measured by NanoDrop (Thermo Scientific) and an equal amount of extracted RNA was reverse-transcribed by SuperScript® III First-Strand Synthesis SuperMix (Invitrogen). cDNAs of NRF2, NQO1 or GAPDH were quantified by real-time PCR (7500 Real Time PCR System, Life Technologies) using TaqMan® Gene Expression MasterMix and TaqMan® Gene Expression Assays for NRF2 (Hs00975961_g1), NQO1 (Hs02512143_s1), and GAPDH (Hs02758991_g1).

### 7. NQO1 gene copy number variation

Genomic DNA was extracted using a PureLink® Genomic DNA Mini Kit (Invitrogen) and quantified by NanoDrop (Thermo Scientific). Equal amounts of genomic DNA were mixed with TaqMan® Genotyping Master Mix supplemented with TaqMan® Copy Number Reference Assay RNase P (Life Technologies) and TaqMan® Copy Number designed to the 1st exon of NQO1 (Hs03028502_cn) or 3' downstream of the NQO1 gene (Hs05457080_cn). Each copy number was quantified by real-time PCR (7500 Real Time PCR System, Life Technologies). NQO1 copy numbers were normalized to the average of normal melanocyte samples as 2 copies.

## Results

### 1. NQO1 expression and 17-AAG sensitivity in melanoma and NSCLC

**Fig 1:**
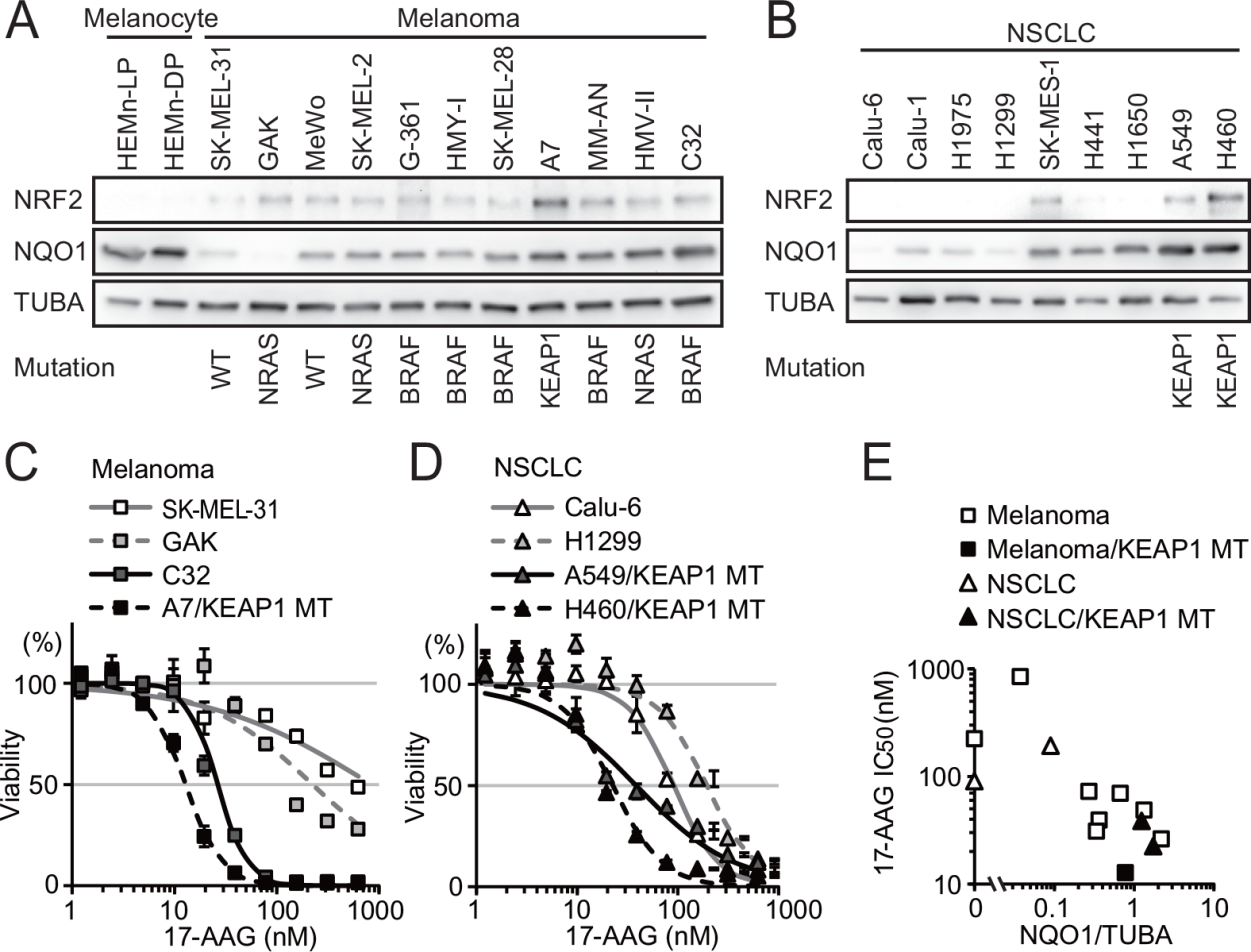
NQO1 expression and 17-AAG sensitivity in melanoma and NSCLC cell lines. (A, B) Expression of NRF2, NQO1 and α-tubulin (TUBA) was detected by immunoblotting analysis in whole extracts of normal melanocytes, melanoma (A), and NSCLC cell lines (B). (C) 17-AAG sensitivity of melanoma cell lines with low NQO1 expression (SK-MEL-31 and GAK) and high NQO1 expression, with and without KEAP1 mutation (C32 and A7, respectively). (D) 17-AAG sensitivity of NSCLC harboring wild-type KEAP1 and NQO1-low (Calu-6 and H1299) and KEAP1-mutated NQO1-high cell lines (A549 and H460). (E) Relationship between NQO1 abundance and 17-AAG sensitivity of melanoma and NSCLC cell lines. The data were expressed as mean±S.D. of four independent experiments (n = 4).

Expression of NRF2 and NQO1 proteins in normal melanocytes, melanoma and NSCLC cell lines with or without KEAP1 mutations was compared by immunoblotting (Fig 1A and 1B). Densitometric quantification of NQO1 signals was normalized against NQO1 signals in normal melanocytes (Table 1). Melanoma cell line A7 and NSCLC cell lines A549 and H460 harbor KEAP1 loss-of-function mutation and express abundant NRF2 and NQO1 in comparison with other cell lines. Surprisingly, normal melanocytes were found to express NQO1 at a level comparable to that in cell lines harboring KEAP1 mutation, whereas NRF2 was not detectable under these conditions (Fig 1A and Table 1). Expression of NRF2 and NQO1 was variable among melanoma cell lines without KEAP1 or NRF2 mutation. Several cell lines were subjected to 17-AAG treatment, and the IC_50_ was calculated. In both melanoma and NSCLC, IC_50_ values for cell lines with KEAP1 mutation and high NQO1 expression were lower than those of cell lines with low NQO1 expression (Fig 1C and 1D). To confirm NQO1-dependent bioactivation of 17-AAG, cells were treated with a NQO1 inhibitor prior to 17-AAG treatment. Pretreatment with the NQO1 inhibitor increased the IC_50_ of 17-AAG by more than 2-fold in all melanoma cell lines tested and in NSCLC cell lines with KEAP1 mutation (Table 1). Correlation analysis revealed a significant inverse correlation between NQO1 abundance and 17-AAG IC_50_ (r = −0.8088, *p* = 0.0008), but not in cells pretreated with the NQO1 inhibitor (r = −0.2971, *p* = 0.3242) (Fig 1E and Table 1).

**Table 1:** NQO1 expression and 17-AAG sensitivity in melanoma and NSCLC cell lines.

	NQO1/TUBA	IC_50_ (nM)	
	densitometry	17-AAG	17-AAG + NQO1 inhibitor
Melanoma			
GAK	0	223.28	564.64
SK-MEL-31	0.04	843.64	4237.35
MeWo	0.27	72.56	473.66
SK-MEL-2	0.34	30.86	98.54
G-361	0.36	39.28	124.12
SK-MEL-28	0.67	69.61	198.41
A7 (KEAP1 MT)	0.78	12.73	49.40
HMV-II	1.33	48.68	123.47
C32	2.19	26.24	399.82
NSCLC			
Calu-6	0	90.38	62.06
H1299	0.09	192.57	347.36
A549 (KEAP1 MT)	1.24	38.45	241.89
H460 (KEAP1 MT)	1.74	22.44	94.69
Spearman's rank correlation between NQO1 and IC_50_			
	Coefficient	−0.8088	−0.2971
	p-value	0.0008	0.3242

### 2. Transcriptional regulation of NQO1 in normal melanocytes and melanoma without KEAP1 mutation

**Fig 2:**
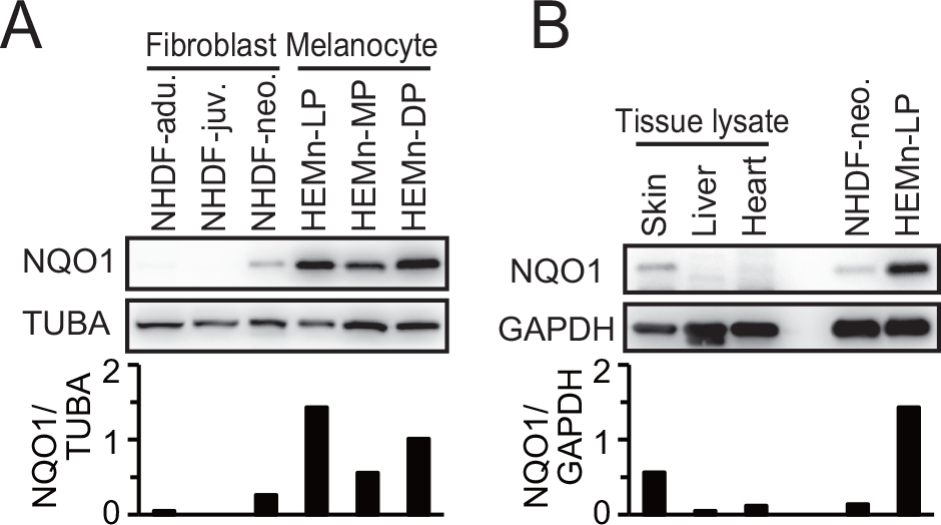
NQO1 expression in normal tissues and primary culture. (A) Normal human dermal fibroblasts (NHDF) from adult, juvenile and neonatal donors, and human epithelial melanocytes (HEM) from light, medium and dark pigmented donors were subjected to immunoblotting to detect NQO1 and TUBA. Each signal was quantified by ImageJ. (B) Protein samples from normal skin, liver or heart were subjected to immunoblotting to detect NQO1 and GAPDH.

**Fig 3:**
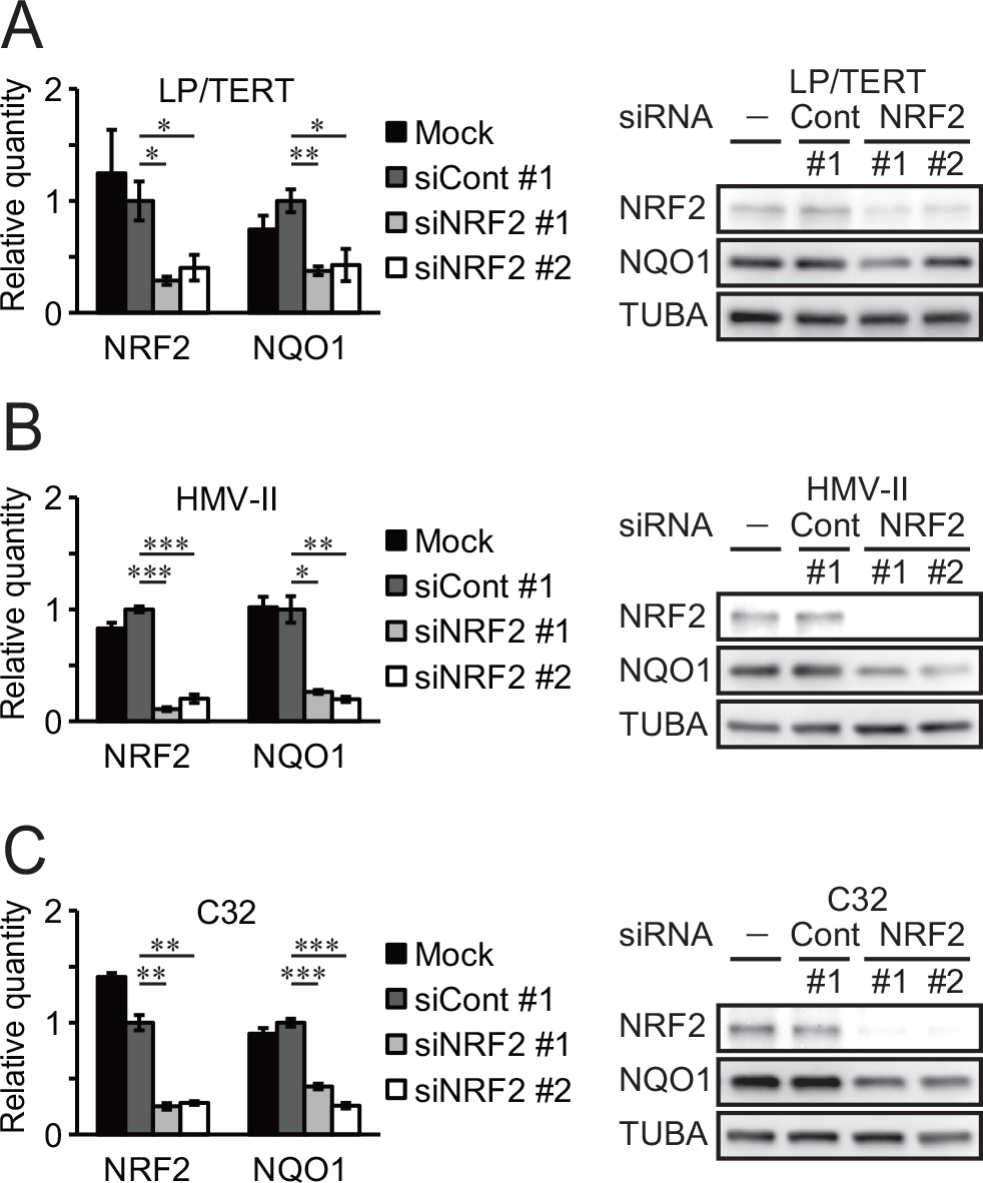
NRF2-dependent NQO1 expression in melanocytes and melanoma cells harboring wild-type KEAP1. (A) Immortalized melanocytes (LP/TERT) were transfected with siRNA against NRF2 or control siRNA (Cont), or treated with transfection medium alone (Mock, −). Total RNA was extracted on day 3 and subjected to RT-PCR to quantify NRF2, NQO1 and GAPDH cDNAs. The data were expressed as mean±S.D. of three independent experiments (n = 3), and statistical significance was expressed as *, *p*<0.05; **, *p*<0.01; and ***, *p*<0.001 compared to control siRNA samples. Whole extracts were prepared on day 4 and subjected to immunoblotting for detection of NRF2, NQO1 and TUBA. (B) HMV-II cells were treated as described in (A), and RT-PCR and immunoblotting were carried out on day 3 and day 6, respectively. (C) C32 cells were treated and subjected to RT-PCR and immunoblotting as described in (B).

NQO1 expression in normal melanocytes was compared with that in normal human dermal fibroblasts. Although NQO1 was detectable in fibroblasts from a neonatal donor, signals in melanocytes were more abundant than those in fibroblasts (Fig 2A). Among normal adult human tissue samples, NQO1 expression was detected in skin but not in liver and heart (Fig 2B). To determine whether NQO1 overexpression in melanoma cell lines harboring wild-type KEAP1 is caused by NQO1 gene amplification, NQO1 copy number variation was analyzed in normal melanocytes and melanoma cell lines. However, NQO1 gene amplification was not detected in melanoma cell lines with wild-type KEAP1 and high NQO1 expression (S1 Fig). In addition, we were unable to find any additional mutations that were associated with KEAP1-NRF2 pathway activation in existing melanoma exome sequence data. We then determined whether NQO1 overexpression is dependent on NRF2 transcriptional activity in melanocytes and melanoma harboring wild-type KEAP1. Immortalized LP/TERT melanocytes and two melanoma cell lines HMV-II and C32 were transfected with siRNA against NRF2 or control siRNA. In all three cell lines, NRF2 siRNA downregulated the expression of both NRF2 and NQO1 at the mRNA and protein levels (Fig 3).

### 3. Synergistic effect of combination treatment with 17-AAG and CDDP in NQO1-low melanoma cell lines

Because either CDDP or DTIC produces ROS, which activate NRF2 and induce NQO1 expression, we determined whether these drugs might sensitize NQO1-low melanoma cells to 17-AAG. NQO1-low melanoma cell lines were treated with a combination of 17-AAG and CDDP or 17-AAG and DTIC at molar ratio of 1:100 or 1:1,000, respectively, and the combination index was calculated according to the Chou-Talalay method. In terms of IC_90_, a synergistic effect of the 17-AAG and CDDP combination was detected in four out of five NQO1-low cell lines (Table 2). In contrast, no synergistic effect was detected in A7 cells, which harbor KEAP1 mutation and express NQO1 constitutively. In terms of IC_50_, the combination treatment with 17-AAG and DTIC exerted a synergistic effect in three cell lines, but the effect became additive or antagonistic at higher toxicity.

**Table 2:** Combination treatment with 17-AAG and CDDP or DTIC.

		Combination index					
	NQO1	17-AAG + CDDP			17-AAG + DTIC		
		IC_50_	IC_75_	IC_90_	IC_50_	IC_75_	IC_90_
SK-MEL-31	−	2.184	1.765	1.444	1.285	1.258	1.291
GAK	−	0.808	**0.664**	**0.593**	0.900	0.961	1.028
MeWo	+	**0.686**	**0.447**	**0.306**	**0.756**	0.955	1.211
SK-MEL-2	+	**0.732**	**0.625**	**0.534**	**0.494**	1.248	4.205
G-361	+	1.176	0.844	**0.666**	1.488	2.702	5.082
A7 (KEAP1 MT)	++	1.275	1.022	0.873	**0.370**	**0.608**	1.172

## Discussion

The present study has clarified that melanoma and NSCLC cell lines showing NQO1 overexpression are sensitive to 17-AAG in comparison with cell lines showing low NQO1 expression. Our previous study identified a KEAP1 frameshift mutation in ~10% of melanoma cell lines and clinical melanoma specimens [10]. In contrast, elevated expression of NQO1 has been reported in several kinds of cancer including melanoma, 30% of primary melanomas showing strong immunopositivity for NQO1 [23]. As observed in the present study, KEAP1 mutation-independent NRF2 activation may be evident in a high proportion of NQO1-high melanomas. Overexpression of NQO1 in KEAP1 wild-type melanoma cell lines and normal melanocyte was dependent on NRF2. We attempted to clarify whether endogenous ROS or the PI3K/AKT pathway might activate NRF2 in KEAP1 wild-type melanoma cells. However, treatment of melanoma cells with the cell-permeable antioxidant N-acetylcysteine, or the PI3K inhibitor LY294002, failed to prevent NQO1 transcription. The participations of other NRF2-activating pathways, such as RAS/RAF activation, GSK3β/Fyn inhibition or overexpression of KEAP1 antagonists [24], remain elusive and will require work. It is noteworthy that NQO1 expression in normal melanocytes was higher than that in dermal fibroblasts and comparable to that in cell lines with KEAP1 mutation. Although events upstream of NRF2 activation are also unclear in normal melanocytes, NRF2 can be maintained in an active state to scavenge naturally occurring ROS during melanin synthesis [25]. Dopaquinone is an endogenous quinone produced in the synthesis of melanin, and is normally present in the melanosome compartment [26,27]. Leakage of dopaquinone or its precursor L-DOPA from melanosomes is thought to be toxic and catechol-O-methyltransferase (COMT) mediates the methylation of cytosolic L-DOPA and prevents its oxidation to dopaquinone [28]. In addition, COMT inhibition increases the abundance of NQO1 [29], which itself modifies melanin synthesis [30], and therefore COMT and NQO1 can complementarily reduce ROS-producing intermediates of melanin synthesis.

We expected that treatment with CDDP or DTIC, either alone or in combination with 17-AAG, would synergistically eliminate NQO1-low melanoma cell lines through induction of NQO1 expression. A synergistic effect was observed in four out of five NQO1-low cell lines treated with a combination of CDDP and 17-AAG, but the results obtained with the DTIC and 17-AAG combination did not support our hypothesis. A similar pharmacological action has been reported in glioma cells [31], and synergism of CDDP with HSP90 inhibitor has also been observed in other types of human cancer [32]. Although HSP90 inhibition or heat shock stress activates HSF1 and induces the expression of a number of molecular chaperones, co-administration of CDDP inhibits HSF1 activation and synergistically reduces the clonogenicity of A549 and HeLa cells [33]. HSF1 knockdown in the melanoma cell line MeWo enhances its sensitivity to heat shock but not to DTIC toxicity [34]. In parallel, the HSP90 client can also take part in CDDP-induced genotoxicity or subsequent apoptotic pathway evasion, such as that involving AKT or IGF1R, or via the JNK-mediated pathway [35,36]. Recently, DNA-PK was identified as a HSP90 client that participates in full activation of DNA-PK during apoptosis, although the function of DNA-PK in combination with a genotoxic agent and HSP90 inhibitor has not been determined [37]. CDDP cross-links intra-strand and inter-strand purine bases, and DNA-PK takes part in the repair of inter-strand cross-links, whereas DNA alkylation by DTIC is repaired by other pathways [38]. Therefore, HSP90 inhibition and DNA-PK dysfunction may exacerbate the genotoxicity induced by CDDP, but not that induced by DTIC.

In conclusion, NQO1 overexpression in melanoma appears to be NRF2-dependent and inversely correlated with 17-AAG sensitivity. Because NRF2 activation is associated with resistance to ROS-based chemotherapy and radiotherapy, 17-AAG could be a potential second-line treatment for NQO1-high melanoma, or in combination with CDDP for NQO1-low melanoma.

## Supporting Information

S1 Data Supporting data for Figs 1 and 3, Tables 1 and 2 and S1 Fig. (XLSX)

S1 Fig NQO1 gene copy number variation in melanoma cell lines. (A) Genomic DNA was extracted from melanocytes and melanoma cell lines, and NQO1 gene copy number was determined using two probes against Exon 1 and the 3' downstream region of the NQO1 gene. Copy number was normalized against normal melanocytes as two copies. The data were expressed as mean±S.D. of three independent experiments (n = 3). (B) NQO1 gene copy number and NQO1 protein abundance were inversely correlated (r = −0.5722) but not to a significant degree (*p* = 0.0697).
(EPS)
